# Nutrient contents of three commonly consumed fruits of lowe’s monkey (*Cercopithecus campbelii lowei*)

**DOI:** 10.1186/s40064-015-0814-0

**Published:** 2015-02-01

**Authors:** Edward D Wiafe

**Affiliations:** Department of Environmental and Natural Resources, Presbyterian University College, P. O. Box 393, Akropong-Akuapem, Ghana

**Keywords:** Proximate analysis, Lowe’s monkey, Nutrition, Monkey fruits

## Abstract

Proximate analysis of three commonly consumed fruits (maize, hog plum and banana) was conducted to determine food biochemistry of the Lowe’s monkeys with specific objectives of determining the nutrient content and chemical composition of the food of Lowe’s monkeys by ‘Weende system of Analysis’. The results indicated the following order of nutrients: Nitrogen-Free-Extract, moisture, protein, fats, ash and fiber. The proportions of the nutrients contents of the three fruits did not differ from each other. This result suggests why frugivorous monkeys do not depend on fruits only but other supplements like insects and arthropods. Further analysis on other food types of the monkey has been recommended.

## Introduction

The population of Lowe’s monkey (*Cercopithecus campbelii lowei*) an endemic upper Guinea primate, classified as Least Concern by the IUCN/SSC (2009), has been observed to be decreasing (Oates, 1999; Deschner and Kpelle, 2003; Gatti, 2010).The monkeys can now be found only in some protected areas, such as Buabeng-Fiema, Ankasa, Bia and Kakum Conservation Areas in Ghana. A more biochemical information is therefore required to enable its conservation in the form of vital information to facilitate the implementation of conservation initiatives such as re-introduction of species to some ‘empty forests’ (Oates, 1999).

Generally, fruits provide animals with more readily accessible nutrients than leaves do. Energy is likely to be especially important although other nutrients may be important in particular cases (Altmann, 1998). But fruits suffer from their own intrinsic disadvantages. Firstly, fruits tend to be more patchily-distributed than leaves and are often highly seasonal in their availability. Secondly, many plants may require primates to swallow their seeds whole (since primates are often a vehicle for seed dispersal) but not to consume seeds before they are matured or to destroy seeds by chewing them into pieces. Hence many plants have evolved defences to protect their seeds from premature dispersal or predation (Cowlishaw and Dunbar, 2000; Waterman *et al*. 1988). Although most primates are vegetarians, most of them also eat small amounts of animal matter which contains vitamin B_12,_ which primates cannot synthesize or obtain from non-animal sources. In most cases, carnivory involves predation on insects and other invertebrates (e.g., worms, small birds and their eggs or nestlings) or small vertebrates like lizards and frogs (Cowlishaw and Dunbar, 2000). In contrast to feeding on small animals, the active hunting of animals as large as ungulates or even medium sized primates is exclusive to chimpanzees (Boesch and Boesch, 1989; Davis and Cowlishaw, 1996).

In order to survive and breed successfully, the animals must obtain adequate food and even where food supplies appear to be abundant, such as in a tropical rain forest, particular components of the diet may be in short supply and competition for these could be intense at certain times of the year. Little information exists on the food items, food availability and factors regulating these for Lowe’s monkeys. However, nutrition is one of the most basic aspects of an animal's ecology and conservation measures such as quantifying suitable habitats, choosing areas for protection or species to be planted for remediation of degraded habitats are possible only if the nutrients of food plants are known. The main goal of this research was to document the biochemistry aspect of the Lowe’s monkeys with specific objective of determining the nutrient composition of the food of Lowe’s monkeys.

The hypothesis has been that the nutrient contents of all the fruits consumed by Lowe’s monkey did not differ from one fruit to another.

## Materials and methods

### Study species

**Figure 1:**
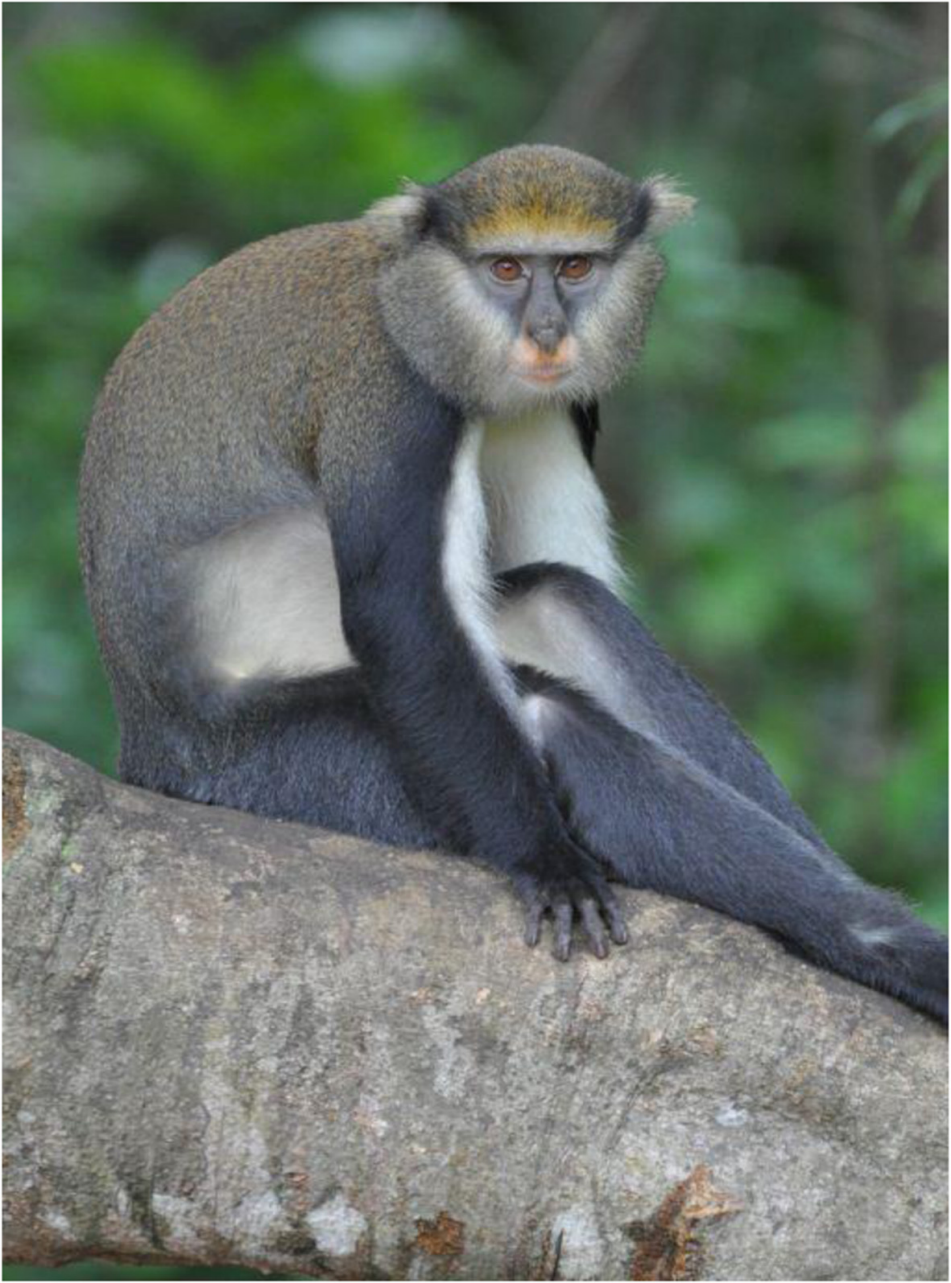
**External morphology of Lowe’s monkey.**

Lowe’s monkey (*Cercopithecus campbelli lowei*) is considered a subspecies of Campbell’s monkey (*Cercopithecus campbelli*) from which two sub-species have been described as *Cercopithecus campbelli campbelli* and *Cercopithecus campbelli lowei*, though the taxonomy is still unresolved. The head and body measure between 40–58 cm, while the tail measures between 54–75 cm. The Lowe’s monkey is a long tailed, arboreal monkey with grizzled brownish black, dark grey hind legs and rump, black outer arms, tail tip, hands and feet (Figure 1). The under parts are white and the finely grizzled cheek fur pales to form a sharp contrast with the blue-grey eye mask that typifies all guenons. A similar narrow margin edges the oval orange-yellow brow band. Ear tufts are grizzled and often yellowish; the temples are marked by a broad black band that separates the light cheeks and ears from dark crown and orange brow (Figure 1). This species is found from River Sassandra (Côte d’Ivoire) to the River Volta (Ghana), in most forest types: primary, secondary and galleries but not common in marshy areas or mangroves. Its food is mainly fruit, pulp of oil palm seeds, figs, cola and garden fruits. It frequently collects flowers and hunts insects but it takes a little interest in other invertebrates (e.g., snail) and leaves (Kingdon, 1997).

### Measurement of nutrient composition of some fruits consumed by lowe’s monkey

The ethics of non-human primate research at UW-Madison was followed.

Nutrient contents of three species that were observed to have been consumed most by the Lowe’s monkeys were analyzed and compared. They were the ripe fruits of banana (*Musa sapientum*) without the peel, fresh matured grains of maize (*Zea mays*) and ripe pulp of Hog plum (*Spondias mombin*).

‘Weende system of Analysis’ or proximate analysis, the most widely used method for determining the composition of feedstuff was used to partition the fruit parts into six fractions: water, ash, crude protein, ether extract (fat), crude fiber and nitrogen-free extract. This analysis was an attempt to simulate animal digestion. After extracting the fat, the sample was subjected to an acid digestion, simulating the acid present in the stomach, followed by an alkaline digestion, simulating the alkaline environment in the small intestine. The crude fiber remaining after digestion was the portion of the sample assumed not digestible by monogastric animals. In the proximate analysis of feedstuffs, Kjeldahl nitrogen, ether extract, crude fiber and ash were determined chemically. The determination of nitrogen allowed the calculation of the protein content of the sample.

Samples of the fruit parts were obtained from KCA, and were subjected to proximate analysis at the Agroforestry Laboratory of the Institute of Renewable Natural Resources, Kwame Nkrumah University of Science and Technology, Kumasi, Ghana.

## Results, analysis and discussions

### Nutrient content of lowe’s monkey food

Table 1 provides the details of the means of three replicates of nutrient content of Hog plum, banana and maize being only three of the fruits observed to be consumed by the Lowe’s monkey. Kruskal-Wallis test indicated no significant difference between the nutrient contents of the three fruits (H = 0.17, *p* = 0.92).

**Table 1:** **Nutrient content of fruits of three food plants commonly consumed by Lowe’s monkeys in KCA**

**Type of Nutrient**	**Maize (%)**		**Banana (%)**		**Hog plum (%)**	
	**Mean**	**SD**	**Mean**	**SD**	**Mean**	**SD**
MOISTURE	8.00	0.00	14.00	0.17	16.03	0.29
ASH	2.00	0.10	2.00	0.00	4.01	0.01
FAT	10.00	0.10	1.05	0.00	5.01	0.00
PROTEIN	11.13	0.04	3.52	0.02	7.88	0.01
FIBRE	1.58	0.02	4.00	0.17	1.02	0.02
NFE	67.04	0.00	75.43	0.38	66.08	0.06

The result of the proximate analysis of fruits of three food plants of Lowe’s monkey indicated that the mean moisture contents of fresh samples of maize was 58% (SD = 3.46, N = 3), banana (68%) (SD = 3.46, N = 3) and hog plum (85.33%) (SD = 1.15, N = 3). Kruskal-Wallis test indicated significant differences among the moisture contents of the fresh samples of the three fruits (H = 7.39, *p* = 0.03). On dry matter basis the study showed that Hog plum contained moisture of 16.03% (SD = 0.29), banana 14.0% (SD = 0.17) and maize 8.0% (SD = 0).Mann–Whitney test indicated a significant difference between moisture content of dried samples of maize and banana (U = 1, *p* = 0.033), maize and hog plum (U = 1, *p* = 0.03) and no difference between hog plum and banana (U = 1, *p* = 0.32).

Nitrogen-free extract (NFE), being an estimate of crude starch and sugar content of a feed, in the three fruits of Lowe’s monkey were as follows: banana contained 75.43% (SD = 0.38), maize 67.04% (SD = 0.00) and hog plum 66.08% (SD = 0.06). The differences in the NFE were significant (H = 7.39, *p* = 0.02) according to Kruskal-Wallis test.

Protein and fat were respectively higher in maize 11.13% (SD = 0.04) and 10.00% (SD = 1.10), relatively more than hog plum of 7.88% (SD = 0.01) and 5.01% (SD = 0.00); and banana of 3.52% (SD = 0.02) and 1.05% (SD = 0.00).

Whereas ash was relatively higher in hog plum 4.00% (SD = 0.01) than maize 2.00% (SD = 0.01) and banana 2.00% (SD = 0.00); fiber was higher in banana 4.00% (SD = 0.17) than maize of 1.58% (SD = 0.02) and hog plum of 1.02% (SD = 0.02). Table 1 presents the summary of the means of the result of proximate analysis of maize, banana and hog plum.

### Nutritional characteristics of the diet of lowe’s monkey

Primates are notably consumers of plants and to a lesser extent animal materials. At the ecosystem level, they also exert a very important feedback control on the vegetation itself and are essential for maintenance of homeostasis of the forest ecosystem (Bourliere, 1985). Lowe’s monkeys at Kakum were observed to have visited fruiting trees whenever those plants bore fruits. Irrespective of the locations of these trees (i.e., both inside and peripheries of the protected area) the monkeys managed to feed on the fruits. The Kruskal-Wallis test of nutrient contents of fruits of three food plants consumed by Lowe’s monkey indicated no statistical difference between them, suggesting that nutrient contents of these three species may be the same. According to Booth (1956) who examined the stomach contents of a few wild specimens, Lowe’s guenons are almost entirely frugivorous. Curtin (2002) also found that the greater part of *Cercopithecus diana roloway’s* food is made up of fruits and the pulp of mature fruits was found to be the most important food category in both dry and wet seasons. This was consistent with Bourliere (1985) that forest monkeys consumed the fleshy part of the fruit rather than the harder stones except the *Chiropotes* spp. This fruit eating habit should not suggest that Lowe’s monkeys are exclusively frugivorous because Bourliere *et al*. (1970) found in Côte d’Ivoire that Lowe’s monkeys eat many flowers, leaves as well as insects, though fruits form greater part of plants observed to have been consumed.

The results of proximate analysis of hog plum, banana and maize (Table 1) indicated that all the fruit pulps consumed by the Lowe’s monkey were high in sugars but low in lipids (fats) and proteins. Although some fruits may be high in a particular mineral, Janson and Chapman (1999) stated that, it seems unlikely that primates would actively seek out such fruits as there is little evidence for taste receptors for minerals other than sodium (Hladik and Simmen, 1997; O’Brien *et al*. 1998). Because of the nutritional deficiencies of fruits as a diet, every predominantly fruit-eating primate complements its diet with either insects or leaves or both (Janson and Chapman, 1999).

Cowlishaw and Dunbar (2000) stated that even though most primates are vegetarians, most eat small amounts of animal matter, which is highly nutritious and contains vitamin B12 which primates cannot synthesize or obtain from non-animal sources. Curtin (2002) found out that the *Cercopithecus diana roloway* spent more time on *Piptadeniastrum africanum* feeding primarily on small immobile insects in the terminal branches; since insect foraging sessions may last more than one hour, and on the same day monkeys may move from one *Piptadeniastrum africanum* to another throughout the day. Bourliere *et al*. (1970) concluded that though the staple food of Lowe’s monkeys is predominantly fruits or vegetables, insects constitute an important part of their diet, probably providing the monkeys with the amino acids essential for growth and reproduction.

Moisture content of nutrients was high in all the three fruits examined in the laboratory (Table 1), which suggests that Lowe’s monkeys obtain a lot of water from their food. This was supported by Bourliere *et al*. (1970) who observed that Lowe’s monkeys seem to drink only sparingly and infrequently and concluded that they obtain sufficient water from their food. This explains why arboreal drinking patterns have been classified as follows: (i) after a shower the monkeys often lick the under part of branches where drops have collected; (ii) monkeys visit certain tree holes where rain water has accumulated, dip in one or the other hand and lick them dry (Bourliere *et al*. 1970).

Furthermore, fruit eating primates have other problems. Firstly, fruits are often chemically defended against insects or mammalian herbivores before the pulp and seed mature, and some continue to be defended even when the pulp is ripe. Secondly, plants have evolved a variety of ways to restrict dispersal of their fruits to a fraction of all of the potential fruit-eating animals in the forest. These include (i) particular fruit presentations (Denslow and Moermond, 1982), (ii) morphologies (Janson, 1998), (iii) ripening schedules (McKey, 1975) and/or (iv) taste of defensive chemicals (Janzen, 1974). This may explain why Lowe’s monkeys use only a fraction of fruit species in the forest, as they are confronted with combination of fruit size, protection, taste, toxicity, inaccessibility, or slow ripening rate. The fruit-eating primates therefore, have to solve the challenge of locating ripe fruit crops that are often sparsely distributed in the tropical forest both in space and time. Moreover, searching for rare fruit trees is likely to be insufficient because detection distances for fruit crops are probably short. Instead, many primates tend to remember the locations of fruit crops over periods of days or weeks, returning at relatively predictable intervals to the same tree crown and moving in relatively straight lines from one resource to the next (Garber, 1989; Janson, 1998). Janson (1998) concluded that spatial memory can increase foraging efficiency up to 300% relative to random searching. Lowe’s guenons in the Kakum Conservation Area invest a lot of energy in jumping from one tree to another. These primates need to develop a strategy each time they wish in reaching the fruits which are held several meters above and or associated insects (personal observation).

## Conclusion and recommendation

The variations in the nutrient contents of fruits of Lowe’s monkeys were found to be statistically not significant. The proximate analysis of the three fruits of Lowe’s monkey showed that the fruits were rich in carbohydrate and moisture more than protein, fat and fibre and would therefore need to supplement the diet with insects which contain high protein and fat contents. This may explain why the Lowe’s monkeys depend on other food items such as insects and leaves as supplement to balance the deficiency in the fruits. It may also be taken as an adaptation to withstand lean seasons when particular fruits become scarce in the ecosystem. Further laboratory analysis should be conducted to ascertain the chemical contents of Lowe’s monkey fruits to determine the levels of anti-nutritional chemicals secreted by the plant. This would help explain the reasons why the monkeys eat the fruits at certain stages and not another.
